# Research on Hereditability of Gastric Cancer in Wuwei City

**Published:** 2006-02

**Authors:** Deng-Hai Mi, Xue-Peng Chen, Hao-Zeng Luo

**Affiliations:** *Gansu Wuwei Tumor Hospital, Wuwei, Gansu Province, China*

**Keywords:** hereditability, gastric cancer

## Abstract

**Aim:**

To explore hereditability of gastric cancer in Wuwei city.

**Methods:**

A case-control study was used to investigate 251 gastric cancer families located in Wuwei city. Li-Mantel-Gan and Falconer regression methods were used to calculate segregation ratios and hereditability.

**Results:**

The segregation ratio in Wuwei families was 0.0774, the hereditability of class I kinfolks and class II kinfolks were respectively 22.91% and 20.07%.

**Conclusion:**

The segregation ratio of gastric cancer in Wuwei was significantly lower than for mono-genetic diseases (0.25), corresponding better with a model of polygenetic diseases. The genetic susceptibility factor accounted for about 1/5-1/4 in the formation of gastric cancer familial aggregation. Mi DH, Chen XP, Luo HZ Research on hereditability of gastric cancer in Wuwei city.

## INTRODUCTION

Wuwei city locates on Gansu Hexi hallway, where its total area is more than 6000 sq. km and its infield area is over 1300 thousand acres. The height of west of Wuwei is from 1800 to 2400 meters above sea level where is an area with farming and herd. The middle part of Wuwei, 1500 to 1800 meters above sea level, is cropper area where it abounds in wheat, rice, corn and yam. East of Wuwei abuts on Tenggeli bigdesert. There are some rivers like Zhamu river, Huangyang river, Jinta river and Xiying river about which its region are all from Qilianin mountain. Total rainfall is about 160 mm and total evaporation is about 2100 mm yearly. Temperature in Wuwei is big difference from seasons, from average -15.40 C in January to 29.10 C in July. The population is about 1000 thousand individual. Mean expectation life-span is 69.07 for man and 68.23 for female in the year 2000. Agriculture population accounts for 83%. There are multi-ethnarch in Wuwei including Han, Man, Hui, Zang, Tu and Weiwuer. Economic level of residents is mostly based on agriculture income. The mean income is about 3000 Yuan a year per people.

The higher occurrence area of gastric cancer is in Wuwei city in northwest of China. Its incidence of gastric cancer was 91.18/100,000 from 2001 to 2002. Prevalent danger factor of Wuwei city from various aspects were studied including environment, biology, chemistry factors like meal, nutrition and nitrosamine content in pickle vegetable. The occurrence and development of gastric cancer might be the results of the environment factor and the host factor interaction. The former researches expressed that it involves the mutation of several oncogenes and anti-oncogenes in the process of the occurrence and development of the gastric cancer. In recent years, it has already obtained some certain achievement to the research on hereditary gastric cancer (1-4). But it needs more studies to finally clarify about prevalent factor of occurrence and death of gastric cancer for Wuwei city, we investigated 251 gastric cancer families from January 2002 to April 2003 in order to open out the heredity pattern and regulations of gastric cancer of the high occurrence region in Wuwei city. The results were reported as follows.

## MATERIALS AND METHODS

### Making sure the core family of gastric cancer proband

Two hundreds and fifty-one gastric cancer probands were from surgical inpatients in Wuwei native place between January 2002 and April 2003. There were 189 male cases and 62 female cases. If a family had 2 cases or more than 2 cases of gastric cancer patients, the recently serious case was taken as proband and core family. The 251 control core families were selected from close neighbors who had no blood relationship with probands.

### Investigation methods

With the specially made inquisition form, the specialized training medical personnels visited probands and the control core families who were made the core to home. It was particularly investigated for their kinfolks of three class (class I, class II and class III), two lines (the patriline and the matriline) and four generations (previous two generation, contemporary and next generation). Class I kinfolks included parent, brethren and family. Class II kinfolks included the grandparents, uncle and aunt. Class III kinfolks included cousin. The investigated contents included general circumstance and case history of gastric cancer.

### Statistics methods

Adopt Li-Mantel-Cart method to estimate the segregation ratio (P) of gastric cancer

Segregation ratio: P=(R-J)/(T-J)
Variance of segregation ratio: SP2=(R-J)(T-R)/(T-J)3
Standard error: SEP=(SP2)1/2
95% confidence interval: P ± 1.96 SEP

T: the total number of brethren; R: the total number of sufferer in brethren; J: the core family number of only 1 sufferer in brethren.

Adopt Falconer regression method to estimate hereditability

Hereditability: h^2^=b/r
Regression coefficient: b= (1-qc) (xc-xr)/ac
Variance: Vb = (1/ac2) (qr/ar2A)
Standard error: Sh=Vb1/2/r
95% confidence interval: h2 ± 1.96sh

r: relative coefficient, xc: difference of susceptibility average and threshold value of proband cases, xr: difference of susceptibility average and threshold value of control kinfolks a: ratio of threshold value probability density and morbidity of general crowd, qr: morbidity of proband cases, qc: morbidity of control kinfolks A: absolute number of proband case kinfolk sufferers.

## RESULTS

### To estimate segregation ratio of gastric cancer

According to the characteristic of case origin and its family, it is listed at Table 1 for distributing of gastric cancer in brethren, and the segregation ratio was estimated. The result revealed that the segregation ratio of Wuwei gastric cancer was 0.0774 and obviously less than 0.25. And its standard error was 0.00006992, 95% confidence interval 0.0609-0.0939. The morbidity of gastric cancer in brethren was far under 1/4 and had significantly difference for that of mono-gene hereditary disease which was less that of dominant inheritance. It was also the same as recessive inheritance. Therefore Wuwei gastric cancer could be polygene hereditary pattern.

**Table 1:** Distributing and estimated segregation ratio of gastric cancer patient in different brethren groups including probands

Brethren number	Family number	Total number of brethren	Total number patient of in brethren	Family number with only one patient in brethren

1	2	2	2	2
2	9	18	4	4
3	48	144	28	26
4	57	228	54	41
5	42	210	45	39
6	49	294	77	51
7	21	147	44	26
8	11	88	20	14
9	9	81	21	13
10	3	30	5	5
Total	251	1242 (T)	300 (R)	221 (J)

P=(R-J)/(T-J)=0.0774, SP^2^=(R-J)(T-R)/(T-J)^3^=0.00006992, SEP=(SP^2^)^1/2^=0.008362.

### To estimate hereditary degree

Hereditability of gastric cancer was listed at Table 2 for in Wuwei city. It was obvious that hereditability of gastric cancer class I kinfolks was 22.91%, male was 22.84% and female was 21.02%, and that of class II kinfolks was 20.07%, male was 26.52%, female was 16.24%. Thus it could be seen that the genetic factor had certain pathogenic function in the outbreak of the gastric cancer.

**Table 2:** Estimated hereditability of class I and class II kinfolks in Wuwei city

Investigated population	Observation people number	Patient number (A)	Prevalence rate (q%)	X	a	b	h^2^ ± sh (%)

Male class I kinfolks of control family	1260	27	2.14	2.030	2.400		
Male class I kinfolks of proband family	1298	52	4.01	1.750	2.153	0.1142	22.84 ± 1.07
Famale class I kinfolks of control family	1112	12	1.08	2.304	2.645		
Famale class I kinfolks of proband family	1145	25	2.18	2.023	2.393	0.1051	21.02 ± 0.93
Control class I kinfolks	2372	39	1.64	2.152	2.509		
Proband class I kinfolks	2443	77	3.15	1.862	2.251	0.1137	22.91 ± 0.72
Male class II kinfolks of control family	1296	17	1.31	2.242	2.589		
Male class II kinfolks of proband family	1335	26	1.95	2.068	2.433	0.0663	26.52 ± 1.74
Famale class II kinfolks of control family	1019	4	0.39	2.662	2.971		
Famale class II kinfolks of proband family	1050	6	0.57	2.541	2.860	0.0406	16.24 ± 1.45
Control class II kinfolks	2315	21	0.91	2.371	2.706		
Proband class II kinfolks	2385	32	1.34	2.234	2.582	0.0502	20.07 ± 1.17

### Gastric cancer family history

It was 20.32% (51/251) that there was gastric cancer family history among probands in case group, and was 9.52% (20/251) in control group, χ^2^=15.99 and *P*<0.01 by χ^2^ test. Considering relative fatalness, class I kinfolks proband was 1.91 times of control, class II kinfolks was 1.47 times of control. χ^2^=11.64 and *P*<0.01 of gastric cancer prevalence rate of class I kinfolks by χ^2^ test, and χ^2^=1.99 and *P*>0.05 of that of class II kinfolks by χ^2^ test that gastric cancer prevalence rate of proband group was higher than that of control group but had not statistics significance. There were 30 ancestry and its ratio was 58.82% (30/51) that gastric cancer patient were more than 3 cases among probands whose family had gastric cancer family history, among there were 25 ancestry and its ratio was 49.02% (25/51) that 1 case was the first kinfolk of other 2 cases, there were 15 cases whose the taken bad age was less than 45.

Being compared segregation ratio and hereditability of gastric cancer with different high occurrence region.

It was listed at Table 3 for segregation ratio and hereditability of gastric cancer at different high occurring region.

**Table 3:** Compared segregation ratio and hereditability of gastric cancer with different high occurring region

Region	Segregation ratio	Hereditability of class I kinfolks

Jiangsu Taixin	0.1206	21.42 ± 1.78
Jiangsu Jintan & Yangzhong	0.0143	23.80 ± 1.96
Gansu Wuwei	0.0774	22.91 ± 0.72

## DISCUSSIONS

It involves the mutation of several oncogenes and antioncogenes in the process of occurrence and development of gastric cancer. In recent years, by the study on hereditary large intestine cancer, Li-Fraumeni symptom and hereditary mammary cancer, ovarian cancer symptom etc. It has been used for reference and promoting function to the study on family gastric cancer. Wuwei city is the high occurrence region of gastric cancer, year incidence rate is more than 59.29/100,000. This has been proved up that gastric cancer has hereditary susceptibility essence and this also makes a reasonable explanation of prevalence character of gastric cancer. By case-control study on gastric cancer, it was found that gastric cancer sufferer who had family history was 20.32% in Wuwei city, prevalence rate of proband gastric cancer was 1.91 times higher than control group. This would indicate that occurrence of gastric cancer had family aggregation character in Wuwei city and that also was found in Yangzhong & Jintan & Taixing of Jiangsu province and Dalian of Liaoling province in the investigation on gastric cancer hereditary factor. Videback and Mosbech found that gastric cancer morbidity that had gastric cancer family history was 4 times of that without family history by follow-up surveying 302 family numbers of gastric cancer sufferer (5). It indicated that hereditary factor had important effect in the process of family gastric cancer occurrence. Hereditary gastric cancer of international collaboration study group (ICG-HGC) constituted diagnostic standard of ICG- HGC about hereditary gastric cancer (6): there were at least 3 cases who were made sure gastric cancer in a ancestry, among 1 case must be the first kinfolk of other 2 cases, and at least consecutive two generation who suffered from gastric cancer, and at least 1 case whose taken bad age was less than 45. By case-control study on gastric cancer in Wuwei city, it was found 30 ancestries had more than 3 cases gastric cancer, among there were 25 ancestries that 1 case was the first kinfolk of other 2 cases, there were 15 cases whose taken bad age was less than 45, these character were similar with HNPCC.

Hereditary disease may be divided into mono-gene and polygene. According to disease colony genetics data of domestic and international in existence, it should be less than 0.25 for segregation ratio of poly-gene hereditary disease. Segregation ratio of Wuei gastric cancer was 0.0774, its hereditary pattern fall short of mono-gene hereditary rule and was polygene hereditary pattern that several genes that had minuteness effect came into being total effect and at last occurred gastric cancer at action of certain environment factors (including carcinogenic epiphyte and nitrosamine in food, chemistry carcinogen, inconsequence meal frame, fruit and truck incepting lack and being short of vitamin C, *et al*. (7)). Generally character of polygene hereditary disease were that the difference of prevalence rate of colony and class I kinfolks related with hereditability, prevalence rate of class I kinfolks was bigger than that of class II kinfolks and that of class II kinfolks was bigger than that of colony. At the data of this text report, prevalence rate of class I kinfolks was 3.15%, class II kinfolks was 1.34%, colony was 1.28%, these accorded with the character of poly-gene hereditary disease.

Hereditability of class I kinfolks that was estimated at this study was 22.91%, and was similar with that being reported by Wu jian-zhong and Sheng jin (1-2), it showed that hereditary factor reacted certainly and about accounted for1/5-1/4 with whole dangerous factor. Whole kin kinfolks of gastric cancer patient may be regarded as being exposed to double influence of hereditary and environment factor, non-kin kinfolk only was exposed to influence of environment factor. Considering hereditary degree of polygene hereditary disease, it was accepted at present that that was counted according to Falconers threshold value theory. Though the influence of environment factor farrago could not completely be excluded, at this study, control group was the same as village, nation, gender, similar with age, and without kin of three generations with proband, the influence of non- hereditary factor was furthest excluded, they made the result accord with fact.

Summarizing above-mentioned discussion, the research on gastric cancer hereditability from macroscopical hereditary epidemiology should distinguish kin and habitation with the influence of gastric cancer. Further considerations are that kin included having blood relationship and common living, having blood relationship and without common living, without blood relationship and having common living. Meanwhile it should open out the occurrence and development of gastric cancer from the molecular biology level and provide scientific gist for etiology prevention of gastric cancer.
